# ICT-based fluorescent probes for intracellular pH and biological species detection

**DOI:** 10.3389/fchem.2023.1304531

**Published:** 2023-12-01

**Authors:** You Wu, Chengyan Ge, Ying Zhang, Yalong Wang, Deteng Zhang

**Affiliations:** ^1^ Institute of Neuroregeneration and Neurorehabilitation, Qingdao University, Qingdao, China; ^2^ The Affiliated Hospital of Qingdao University, Qingdao, Shandong, China; ^3^ State Key Laboratory of Digital Medical Engineering, School of Biomedical Engineering, Hainan University, Haikou, China

**Keywords:** intramolecular charge transfer, fluorescent probes, intracellular pH, gases, metal ions, anions

## Abstract

Fluorescent probes, typically based on the intramolecular charge transfer (ICT) mechanism, have received considerable research attention in cell detection due to their non-invasiveness, fast response, easy regulation, high sensitivity, and low damage tolerance for *in vivo* bio-samples. Generally, intracellular pH and biological species such as various gases, metal ions, and anions constitute the foundation of cells and participate in the basic physiological processes, whose abnormal level can lead to poisoning, cardiovascular disease, and cancer in living organisms. Therefore, monitoring of their quantity plays an essential role in understanding the status of organisms and preventing, diagnosing, and treating diseases. In the last decades, remarkable progress has been made in developing ICT probes for the detection of biological elements. In this review, we highlight the recent ICT probes focusing primarily on the detection of intracellular pH, various gases (H_2_S, CO, H_2_O_2_, and NO), metal ions (Cu^2+^, Hg^2+^, Pb^2+^, Zn^2+^, and Al^3+^), and anions (ClO^−^, CN^−^, SO_3_^2−^, and F^−^). In addition, we discuss the issues and limitations of ICT-based fluorescent probes for *in vivo* detection and explore the clinical translational potential and challenges of these materials, providing valuable guidance and insights for the design of fluorescent materials.

## Introduction

Fluorescent probes have been regarded as an easy and powerful approach in biological fields such as cell imaging, biosensors, and early cancer diagnosis at the molecular level due to their rapid response, specificity, real-time capability, and high reactivity both *in vitro* and *in vivo* (Danylchuk et al., 2021). Generally, fluorescent probes are mainly composed of a fluorophore group and a recognition group, which are bridged by a linker. The intensity of emission wavelength could be tuned by the interaction between targets and reactive recognition groups, normally based on the mechanisms including fluorescence resonance energy transfer (FRET), intramolecular charge transfer (ICT), twisted intramolecular charge transfer (TICT), photo-induced electron transfer (PET), aggregation-induced emission (AIE), and through-bond energy transfer (TBET) (Fang et al., 2023). Typically, ICT is one of the traditional sensing mechanisms, and ICT-based fluorescent probes have been obtained and applied in organisms due to their biocompatibility, good stability, tunable spectrum, and easy processability (Jana et al., 2021). TICT fluorescent probes usually exhibit low fluorescence efficiency in solution but show significantly increased fluorescence emission intensity in specific environments such as inside organisms due to TICT. Therefore, TICT fluorescent probes have high sensitivity in detecting microenvironment changes and molecular conformational changes within biological systems. PET fluorescent probes are usually composed of a fluorophore and an electron acceptor, and their fluorescence can be switched on/off through the PET process. When the electron acceptor binds with the target molecule, the PET process is affected, leading to changes in fluorescence emission intensity. This property allows PET fluorescent probes for use in quantitative and positional analyses of biomolecules. AIE fluorescent probes emit weak or no fluorescence in dilute solutions but exhibit strong fluorescence in high concentrations or aggregated states. This aggregation-induced emission phenomenon makes AIE fluorescent probes advantageous in applications such as biological imaging and biosensors, with features including high sensitivity, low background signal, and excellent optical properties. TBET fluorescent probes modulate fluorescence through energy transfer in the form of bond energy transfer. When bond energy transfer occurs in specific environments, the energy state of the fluorophore changes, resulting in changes in fluorescence emission. TBET fluorescent probes can be used to detect molecular interactions, changes in molecular structure, and environmental changes inside cells. However, ICT fluorescent probes have diverse and highly sensitive characteristics for detecting biological elements through different mechanisms and regulation strategies. They have wide application potential in the field of biomedical imaging, drug delivery, and molecular recognition and are expected to play important roles in biomedical research and clinical diagnostics.

**FIGURE 1:**
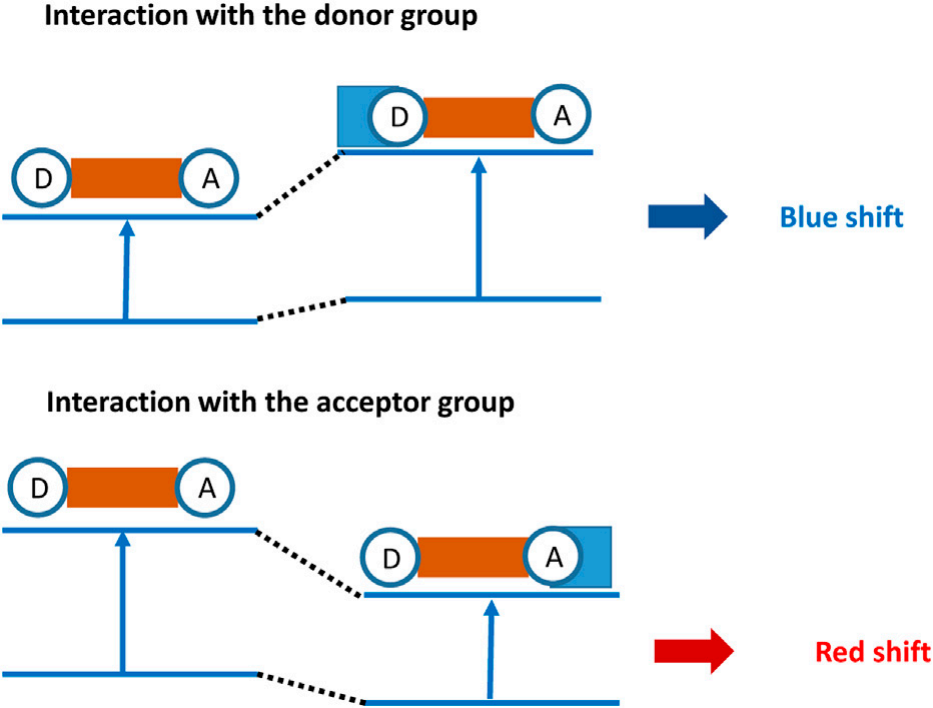
Principle of recognition for fluorescent probes based on ICT.

ICT-based fluorescent probes have a donor–acceptor structure, which are connected together through conjugated function, forming an electron system with the push–pull effect (Jana et al., 2021; Huang et al., 2023). This contributes to the charge in the whole dye system and shows a certain distribution structure. Thus, the formed ground-state electron cloud in the dye determines the fluorescence spectrum performance. When the fluorescent probe undergoes coordination with the analyte, the energy gap between the HOMO and LUMO orbitals of the fluorophore is altered. Due to the different binding affinities between the analyte and the fluorescent probe, the wavelength can experience a redshift or blueshift (shown in Figure 1) (Peng et al., 2021). Redshift refers to the increased electron-withdrawing ability of the fluorophore upon binding to the analyte, leading to an increase in electron cloud density and a decrease in the energy gap between the HOMO and LUMO orbitals, resulting in a longer wavelength. Conversely, blueshift refers to a decrease in wavelength as a result of the electron-donating ability. These probes exhibit a shift in the spectrum wavelength, either redshift or blueshift, upon the addition of the analyte. The original fluorescence intensity decreases, while a new fluorescence intensity gradually increases. Therefore, by utilizing the ICT mechanism, ratio-based fluorescent probes can be constructed to achieve selective detection of the analyte (Jana et al., 2022).

Cells are the basic units that constitute living organisms and carry out life activities. They rely on intracellular pH and biological species such as various gases, metal ions, and anions to function normally in a highly complex and networked spatial environment in order to maintain normal cellular activities (Klymchenko, 2022). When these molecules exhibit an abnormal behavior, it can impair cell function and ultimately lead to the development of diseases in the organism (Pramanik and Das, 2021). For example, thiols play a very important role in the redox homeostasis within cells. Imbalance in the redox state can lead to changes in the cellular metabolism, including respiration. Hydrogen sulfide (H_2_S) is considered to participate in various physiological processes. It can be overexpressed in many cancer cells, and colon cancer cells can produce a large amount of H_2_S through excessive expression of cystathionine β-synthase (CBS). Therefore, high-sensitivity detection of small molecules and biomacromolecules in cells is of great significance for the understanding of the molecular-level cellular functions and the mechanism underlying disease development. Herein, we reviewed the recent developments of ICT probes focusing primarily on the detection of intracellular pH, various gases (H_2_S, CO, H_2_O_2_, and NO), metal ions (Cu^2+^, Hg^2+^, Pb^2+^, Zn^2+^, and Al^3+^), and anions (ClO^−^, CN^−^, SO_3_^2−^, and F^−^). Additionally, the issues and limitations of ICT-based fluorescent probes for *in vivo* detection were discussed, and the clinical translational potential was explored.

### Intracellular pH detection

The intracellular pH (pHi) plays an irreplaceable role in maintaining various metabolic processes in cells. Enzyme activity, signal transduction, and other cellular functions are closely related to pHi. Studies have shown that various diseases, including cancer, Alzheimer’s disease, and Down syndrome, are closely associated with abnormal pHi in cells. Therefore, real-time, accurate, and sensitive monitoring of intracellular pHi changes reveals important information for studying the pathological and physiological processes related to cellular pHi. Small-molecule fluorescent probes used for measuring the pH of specific compartments within cells usually require the following characteristics: 1) the probe must have the ability to target the specific compartment; 2) the linear response range of the probe must include the pH value of the compartment, and its pKa should be as close as possible to the pH value of the compartment to achieve maximum sensitivity; 3) the probe should have a strong anti-interference ability against various cations, amino acids, and other biomolecules present in the cell; 4) reversibility is necessary for the real-time monitoring of pH fluctuations using the probe; and 5) the probe should have low toxicity to cells, excellent membrane permeability, and leak-proof ability. For instance, a ratio-type fluorescent probe, benzo (1,2-b:4,5-b′)dithiophene (BTDB), with a large Stokes shift was prepared based on the ICT luminescence mechanism (Zhang et al., 2016b). BTDB emits at 425 nm under alkaline conditions. As the pH decreases, the N on the aniline group becomes protonated, which enhanced the ICT effect and made the emission band gradually shift to 595 nm (a Stokes shift of 177 nm), presenting excellent lysosome-targeting ability in cells. In another research, a benzoindole-based colorimetric and naked-eye hemicyanine fluorescent probe 2,3-trimethy-3-[2,4-(dihyoxyl-4-yl)vinyl]-3H-benzo[e]indole (BiDD) was obtained for pH detection within the range of 4.4–6.2. In addition, BiDD also possessed high sensitivity and selectivity and low cytotoxicity in HeLa cells when the fluctuations in pH were visualized (Zhang et al., 2020).

### Gas detection

**FIGURE 2:**
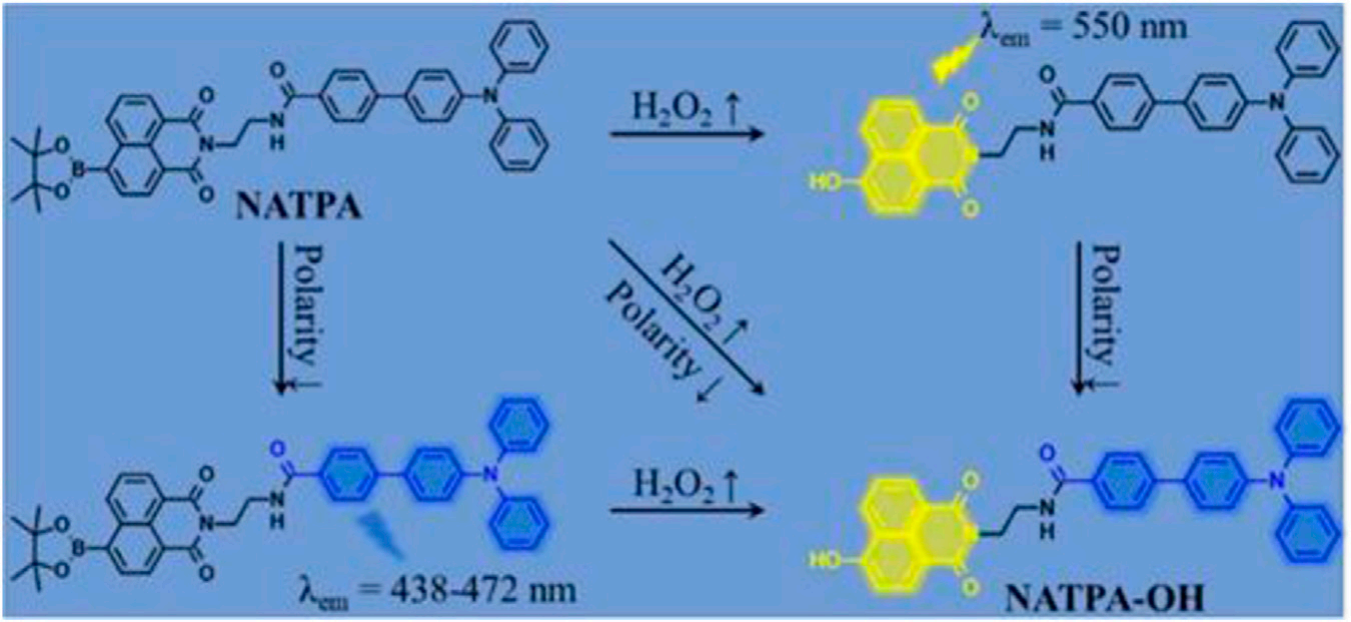
Design strategy of a dual-site fluorescent probe NATPA.

Normally, abnormal concentrations of cellular gases such as H_2_S, CO, H_2_O_2_, NO, and SO_2_ always lead to serious diseases, including ischemia–reperfusion injury, vasodilation, cell death, angiogenesis, neural regulation, inflammation therapy, insulin signaling, and gas stress response (Liu et al., 2023a). Niu et al. (2022) introduced nitrobenzoxadiazole (NBD) ether and NBD amine as thiol recognition sites into the fluorescent probe for H_2_S detection as low as 81.1 nM. Excessive CO can cause oxygen deprivation in organisms, even posing a life-threatening risk. For CO detection, the fluorescent group naphthalimide, based on nitro-to-amino conversion, showed dazzling green fluorescence (Zhang et al., 2021). Furthermore, biological imaging experiments demonstrated that the ICT-based probes could control the changes in exogenous/endogenous CO in living cells. Excessive H_2_O_2_ can induce inflammatory responses, impair the microenvironment of tissue regeneration, and hinder wound healing. Li et al. (2022) first fabricated a naphthalimide−triphenylamine-based probe for H_2_O_2_ imaging with a low concentration of 44 nM and a quick reaction time (15 min) to H_2_O_2_ upon ICT effect in HepG2 cells (shown in Figure 2). NO participates in various physiological and pathological processes, whose homeostasis is related to cardiovascular diseases. A dicarboximide anthracene-based fluorescent probe was constructed to detect the NO level in living cells, showing an excellent sensitivity (5.52 nM) to NO in zebrafish (Wang et al., 2022). In addition, epidemiological research proved that inhalation of SO_2_ can harm the nervous system through destroyed redox balance in cells. Liu et al. (2023b) reported a dual-site multifunctional coumarin fluorescent probe for H_2_O_2_ and SO_2_ (derivatives SO_3_^2−^) detection *in vitro* and *in vivo*.

### Metal ion detection

Metal ions, including Cu^2+^, Hg^2+^, Pb^2+^, Zn^2+^, and Al^3+^, can cause chronic poisoning when they accumulate to a certain concentration (Alizadeh et al., 2020). They can accumulate thousands of times in organisms, causing significant harm to the nervous system, digestive system, immune system, kidneys, and liver. In addition, toxic metal ions can accumulate in the body and cause harm to living organisms even at low concentrations (Alizadeh et al., 2020; Zhao et al., 2023). In addition, heavy metal ions can affect the activity of DNA and proteins, causing chronic toxicity. Therefore, exploring a sensitive and rapid method for detecting heavy metal ions is of great significance and urgency for human safety. Hence, an ICT-based fluorescent probe (E)− 2-{3-[4-(diethylamino)phenyl]acryloyl}phenylpyridinecarboxylate (DPAP) was designed and obtained for the specific detection of Cu^2+^, whose detection limit was as low as 15.2 nM with low cell toxicity and good permeability (Liu et al., 2023c). In another work, a malononitrile isophorone-based probe was fabricated to detect Hg^2+^ rapidly with low biological toxicity. It also possessed a larger Stokes shift and a pronounced UV-vis absorption redshift, suggesting the great potential of Hg^2+^ detection in living cells (Pei et al., 2023). Moreover, (E)-N 0 -{[2-(4′-(diphenylamino)-(1,1′-biphenyl)-4-yl)-2H-1,2,3-triazol-4-yl]methylene} (DBTBH) was synthesized for Pb^2+^ detection with a limit concentration of 4.49 × 10^−8^ M in HeLa cells, showing a promising platform for biological applications (Xue et al., 2021). In addition, a dual fluorescence probe 7-hydroxy-8-{[(2-(hydroxymethyl)quinolin-8-yl)imino]methyl}-coumarin (XL), which consists of formylcoumarin and aminoquinoline moieties, was obtained for Zn^2+^ and Al^3+^ ion detection in PC12 cells, with detection limits of 3.75 × 10^−8^ and 1.14 × 10^−8^ M, respectively (Fu et al., 2020).

### Anion detection

Anions in cells such as ClO^−^, CN^−^, SO_3_^2−^, and F^−^ play important roles in cell proliferation, cell differentiation, immune response, energy conversion, and signal transduction (Zheng et al., 2023). They exhibit high reactivity and can directly modify various biomolecules through oxidation, nitration, and halogenation. Recently, a ClO^−^ probe was reported, which consists of N-alkyl-1,8-naphthalimide-4-boronate ester (NPI) and rhodamine B linked by ethylenediamine. Then, the boronate ester was grafted to the compound to react with HClO at 1.36 nM (Zhu et al., 2023). Next, a CN^−^ probe was designed and synthesized based on a fluorene group and a hemicyanine group, which were linked by a conjugated linker. The probe was found to present rapid reaction, high sensitivity, and selectivity with CN^−^ (Li et al., 2018). In addition, a benzimidazole-based fluorescence probe was synthesized for the detection of SO_3_^2−^. Prior to the addition of SO_3_^2−^, the probe is excited by light, and the electrons from the electron-donating benzimidazole moiety are transferred to the electron-accepting aldehyde moiety, resulting in an ICT effect. However, upon the addition of Na_2_SO_3_, the probe undergoes a nucleophilic addition reaction, inhibiting the ICT effect and making the green fluorescence change to blue. This process was certificated by mass spectrometry, hydrogen spectroscopy titration, and theoretical calculations. Finally, the probe was employed for the sulfite detection in HepG2 cells (Zhang et al., 2016a). Last but important is the F^−^ fluorescent probe which utilizes an F-triggered specific demethylation reaction to induce the opening of the intramolecular charge transfer (ICT) interaction between the donor phenolate anion on IS-NR-O and the acceptor acetonitrile, thereby achieving a dual colorimetric and fluorescent response to fluoride ions with excellent selectivity. Furthermore, the probe exhibits a good linear relationship with fluoride ions over a wide concentration range of 0.38–6.84 ppm and a low detection limit of 0.09 ppm (Hu et al., 2019).

## Conclusion

Recently, various fluorescent probes based on ICT have been designed and gained for intracellular pH and biological species detection, which was believed to promote the developments in applications of biomedicine. In summary, through the design and modification of ICT fluorescent probes, biological targets can be detected accurately. Ratio-type fluorescent probes with a large Stokes shift can dynamically monitor changes in intracellular pH in real time. By designing the D-A structure, different types of ICT fluorescent probes can be used for the rapid detection of various gases, heavy metal ions, and anions in living organisms at the nM level.

However, there are still several aspects that need to be further explored. These include the following: 1. High sensitivity. Reducing the effective concentration threshold of the fluorescent probe improves its sensitivity, which was at lower concentrations of reactive groups, resulting in better detection results. 2. Specificity and selectivity. Fluorescent probes should be able to recognize and bind to target molecules or pathological changes while not interacting non-specifically with other molecules or cells. It is challenging to improve the specific detection of fluorescent probes and reduce non-specific signal interference to obtain better imaging results. It is also promising to cleverly design and synthesize probes to achieve the detection of multiple biological activities with a single probe, thereby achieving multiple detection biological species. 3. Biocompatibility. It is a basic requirement to ensure the safety to the human body and compatibility with biological tissues in the conversion process of fluorescent probes. Fluorescent probes must have low toxicity, low immunogenicity, and good biocompatibility in order to be safely used in clinical applications. The metabolic pathways and toxicity of synthesized molecular compounds must be clarified clearly in organisms, especially for probes that are bound to high-molecular-weight polymers. The metabolic pathways of degradation products and inflammatory reactions also need to be investigated in organs such as the heart, liver, spleen, lungs, and kidneys. 4. Cell permeability. Fluorescent probes should own the ability to pass through the cell membrane and reach target cells or tissues. This requires fluorescent probes to have appropriate chemical properties and size in order to effectively penetrate and distribute in the organism. 5. Integrated diagnosis and therapy. It is a promising solution to integrate the detection of fluorescent probes with treatment by combining them with drugs or nanomedicine delivery systems, to achieve integrated diagnosis and therapy.

More importantly, how to translate laboratory findings into clinical applications and successfully achieve the clinical translation of fluorescent probes is a challenging task that needs to be urgently addressed. Moreover, fluorescent probes need to maintain satisfying stability in the *in vivo* environment, unaffected by factors such as light, acidity or alkalinity, and temperature. This is crucial for achieving reliable fluorescent signals in clinical applications. In addition, fluorescent probes must have good optical properties such as high fluorescence quantum yield, long fluorescence lifetime, and high photostability. This can enhance the intensity and stability of fluorescent signals, making them more suitable for medical imaging or diagnostics. Effectively labeling fluorescent probes onto suitable carriers or nanoparticles and delivering them to target cells or tissues is also a challenging task. More importantly, converting fluorescent probes into clinical applications requires conducting clinical trials to validate their safety and efficacy. Additionally, they need to comply with relevant regulatory requirements and regulations. These challenges require continuous research and development to drive the translation of fluorescent probes from the laboratory to the clinic. Interdisciplinary collaboration and technological innovation will play a crucial role in overcoming these challenges.
